# Paul Hoffmann and the cutaneous silent period test

**DOI:** 10.1055/s-0045-1806826

**Published:** 2025-06-01

**Authors:** Otto Jesus Hernández Fustes, Cláudia Suemi Kamoi Kay, Paulo José Lorenzoni, Renata Dal-Prá Ducci, Paula Raquel do Vale Pascoal Rodrigues, Rosana Herminia Scola

**Affiliations:** 1 Universidade Federal do Paraná, Complexo Hospital de Clínicas, Departamento de Clínica Médica, Serviço de Neurologia, Serviço de Doenças Neuromusculares e Desmielinizantes, Curitiba PR, Brazil.

**Keywords:** Cutaneous Silent Period, Neurology, Neurophysiology

## Abstract

We herein present aspects of the biography of Professor Paul Hoffmann, covering his main works, mainly the article describing the cutaneous silent period, a new technique when it was published in 1922, with a review of its importance, its role in clinical neurophysiology, and its clinical application.

## INTRODUCTION

In 1922, German physiologist Paul Hoffmann described the basis for a neurophysiological test that enables the assessment of the integrity of the structures of the spinal cord and of the A-delta small sensory fibers. His monograph *Untersuchungen über die eigenreflexe (sehnenreflexe) menschlicher muskeln* (“Investigations into the self-reflexes [tendon reflexes] of human muscles) was published by Springer in 1922. 1

Best remembered for the description of the H reflex, a monosynaptic reflex pathway whereby stimulated afferent, sensory nerves synapse with anterior horn cells within the spinal cord and subsequently activate muscle fibers innervated by these motor units, 2 or by the sign that bears his name, described in March 1915, in “Concerning a method for assessing the success of a nerve suture”. 3

He described the sign used to assess nerve regeneration, which was checked by lightly percussing with an extended finger in an area located over the proximal nerve stump or by percussing distally to proximally along the segment of nerve injury or repair. The sign was considered positive if percussion at these sites caused a tingling phenomenon, which radiated in the sensory distribution of the peripheral nerve, known as *Hoffmann and Tinel sign* . 2 4

With the postulate of a monosynaptic transmission, Hoffmann made a significant contribution to the elucidation of functional principles in the central nervous system. This also applies to the interpretation of the postreflective, temporary decrease in the tonic basic innervation, which he interpreted as the inhibitory reflex of the spinal cord and which later received great attention under the term *silent period* , describing exteroceptive suppression, by the effect of muscle contraction during volitional electromyography (EMG) activity.

## THE CUTANEOUS SILENT PERIOD TEST

The cutaneous silent period (CSP) is the transient suppression of continuous EMG activity induced by sensory nerve stimulation. It has been more studied in hand muscles after electrical stimulation of finger nerves, but other recording sites have been used as well. Since the sensory stimuli that mediate the CSP are transmitted mainly by slow-conducting A-delta-type nociceptive fibers that reach the posterior horn of the medulla (laminae of Rexed I and V) and produce pre- or postsynaptic inhibition, from motor neurons, transmitted through of interspinal motor neurons, it has been suggested that the CSP can be considered part of a global detrimental response. 5 The functional significance of the CSP may be to prepare the limbs to quickly move away from an attacking object, preferentially inhibiting the muscles that mediate reaching and grasping while enabling the activation of the proximal muscles that retract the limb. 6

It has been proposed that the CSP may be useful in neurophysiological assessment to investigate spinal nociception, sensorimotor integration, and motor control. Since the CSP has been found to be impaired in several disorders of motor control, it seems conceivable that the interneuronal substrate mediating CSP represents the final common pathway subservient to several aspects of motor control. 6

The clinical usefulness of the CSP depends on the possibility of evaluating proximal segments and components of sensory nerves that are not assessed by conventional neuroconduction studies. In a comprehensive review divided in two parts, Kofler et al. 7 8 (2019), examining the literature from the last 25 years on pathophysiological conditions, reported that the CSP may have a diagnostic role in the evaluation of small-fiber neuropathies, in particular axonal polyneuropathies that affect purely or predominantly fibers with small diameters. Furthermore, in central nervous system diseases, the most useful clinical application of the CSP appears to be the functional diagnosis of intramedullary lesions, such as tumors, and myelopathy. 7 8

We agree with Serrao 9 (2019) that the CSP is a potentially underused tool in clinical neurophysiology. 9 The CSP has been found to be abnormal in several other diseases involving the peripheral and central nervous systems. However, as Kofler et al. 7 8 state, small population samples and lack of test-retest reliability represent limitations that will need to be addressed in future studies.

## PAUL HOFFMANN

Paul Hoffmann was born on July 1st, 1884, in Dorpat, Russia (currently called Tartu, and located in Estonia). His father Friedrich was a professor of internal medicine in Dorpat. 2

He studied medicine in Berlin and Leipzig, where he received his doctorate in 1909. After 2 years as an employee of the Berlin University, he became an assistant at the Physiological Institute of the University of Würzburg in 1911 and completed his habilitation there in 1912 under Maximilian von Frey.

As early as 1912, Paul Hoffmann received his habilitation with his thesis on “The flow of action of the muscle poisoned with Veratrin”. The most fruitful time of his scientific life were the years from 1909 to 1922 in Berlin and Würzburg.

In 1910, Paul Hoffmann published the fundamental work on muscle reflexes as “Contributions to the knowledge of human reflexes with special consideration of electrical phenomena”. In it, he described the direct and the reflex response in the triceps surae muscle in humans after electrical stimulation of the tibial nerve in the popliteal fossa.

Paul Hoffmann was appointed Professor of Physiology at the Freiburg Faculty of Medicine in 1924, where he succeeded Johannes von Kries, the well-known sensory physiologist.

He came to Freiburg as a relatively young professor, where he met many well-known scientists, some of whom later received the Nobel Prize, such as Wieland in 1927 for chemistry, and Spemann in 1935 for medicine and physiology. Paul Hoffmann remained in Freiburg until his retirement in 1955, dying in 1962 of a heart attack. 2

In conclusion, in 1950, J.W. Magladery, from Baltimore, in 1950 named H-Reflex in honor of Paul Hoffmann. 2 He has received an honorary chair from the Universidade de Santiago de Compostela, in Spain, and an honorary doctorate from the universities of Zurich and Berlin, as well as the Hereditary Medal of the German Neurological Society. 2 Some authors have described him as the “originator” of modern neurophysiology in Germany.

Despite the limited amount of articles, we agree with Kofler et al., 7 8 who showed that the potential clinical usefulness of the CSP depends on the possibility of evaluating segments and components of sensory nerves that are not exhaustively evaluated by standard electrodiagnostic methods. The CSP may have a diagnostic role in the evaluation of small-fiber neuropathies, diseases of the central nervous system, and the functional diagnosis of intramedullary lesions.

**Figure 1:**
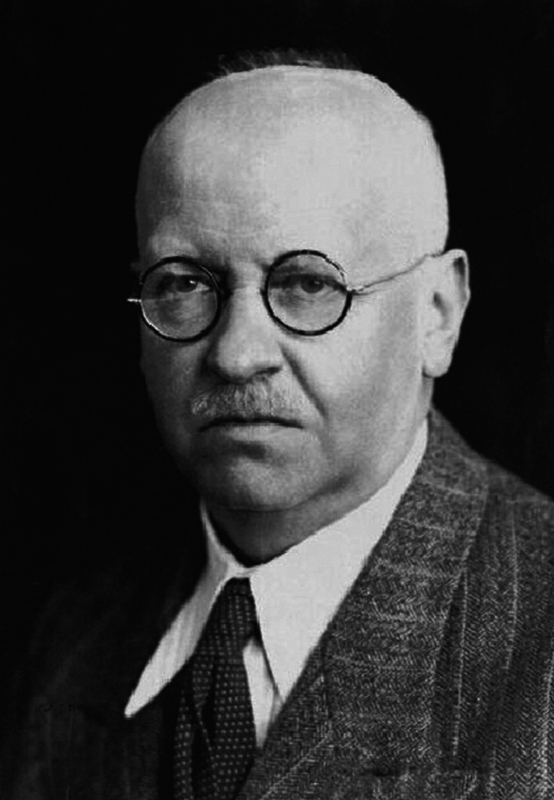
Source: Wikipedia. Paul Hoffmann.
